# Challenges in applying the short Childbirth Self-Efficacy Inventory (CBSEI-C32) in German

**DOI:** 10.18332/ejm/136453

**Published:** 2021-06-18

**Authors:** Laura A. Zinsser, Gaby Schmidt, Kathrin Stoll, Mechthild M. Gross

**Affiliations:** 1 Midwifery Research and Education Unit, Hannover Medical School, Hannover, Germany; 2 Birth Place Lab, Department of Family Practice, Faculty of Medicine, University of British Columbia, Vancouver, Canada

**Keywords:** childbirth self-efficacy inventory (CBSEI), translation, health, midwifery, birth, coping

## Abstract

**INTRODUCTION:**

This study aimed to review and pilot-test feedback from childbearing women who completed the German short version of the Childbirth Self-Efficacy Inventory (CBSEI-C32), which is widely used and validated in different languages.

**METHODS:**

Ten pregnant nulliparas, who planned a natural childbirth, completed the German CBSEI-C32 and provided comments about the comprehensibility of the tool.

**RESULTS:**

When applying the standardized translated German CBSEI-C32, we discovered that women generally gave positive feedback, and reported that the items made them think about coping strategies for labor and birth. Some pregnant woman had problems in understanding two items: ‘Mich beherrschen’ (original English item: ‘Keep myself in control’), and ‘Mich ruhig halten’ (original English item: ‘Keep myself calm’). Some of the items were not comprehensible for pregnant women and might not represent contemporary concepts of childbirth self-efficacy.

**CONCLUSIONS:**

Two items of the German CBSEI-C32 were interpreted ambiguously by the pilot testers. The CBSEI should be checked to identify which items could serve as the basis for a new questionnaire because there are clear and appropriate coping strategies when dealing with labour pain such as item 3 on breathing. These could be complemented with other coping behaviours that are positively worded and serve to empower rather than restrain women. For measuring self-efficacy beliefs in childbirth nowadays, it appears that health-oriented aspects, such as concentrating on the pauses between contractions or mentally staying in the present moment, are more important for women than focusing on control during childbirth.

## INTRODUCTION

### Self-efficacy in childbirth

Childbirth self-efficacy beliefs refer to confidence in one’s abilities to cope with childbirth^1,2^. Self-efficacy is part of the health-oriented concept of salutogenesis and is identified as a health resource, based on its association with performance and coping^1^. When faced with difficult situations, people with lower self-efficacy have higher levels of anxiety and self-doubts, and they try to avoid challenging environmental demands, compared to people with higher self-efficacy scores^3^. Research with childbearing women has shown that high childbirth self-efficacy is related to a reduced likelihood of requesting a caesarean birth^4^, reduced prenatal anxiety, and reduced need for pain management during labor and birth^5,6^. It is also positively associated with emotional wellbeing during pregnancy^7^. To measure self-efficacy, researchers have developed context-sensitive scales. Apart from a general self-efficacy score^8^, there are, for example, scales for pain self-efficacy^9^, parenting self-efficacy^10^, breastfeeding self-efficacy^11^ and childbirth self-efficacy^2^.

### Development of the Childbirth Self-Efficacy Inventory

Lowe^2^ created a comprehensive tool to assess childbirth self-efficacy which is called Childbirth Self-Efficacy Inventory (CBSEI). The inventory contains 16 statements which were first introduced in 1993. It was developed based on the experience of 23 nulliparas and 25 multiparas who gave birth spontaneously. Within 48 hours postpartum semi-structured interviews were carried out. The strategies that women used to cope with labor and birth were categorized into nine behaviors: thinking, concentration/distraction, support, control, breathing, relaxation, emotive, self-encouragement, and uncategorized. Four experts (with expertise of self-efficacy theory and/or childbirth) evaluated the items. The category emotive, which includes for example, screaming, getting angry etc. was excluded. Finally, 16 items were pilot tested with 76 women, followed by the application of the measure to a larger sample of 287 nulliparas, and 95 multiparas (n=382)^2^. The final CBSEI is divided into outcome expectancies (OE, how helpful one believes the behavior to be) and efficacy expectancies (EE, how confident one is that one can enact the behavior). Each set of questions for OE and EE were asked for the first stage (15 questions) and second stage (16 questions) of labor, for a total of four scales. The CBSEI is widely used and has been translated into different languages, like Chinese, Farsi, Arabic, Swedish, Greek, and German^5,12-17^. A short version exists with a total of 32 questions for the second stage of labor and birth (CBSEI-C32)^5,17^. This leads to a reduction of complexity of the CBSEI, for the user^13,17^. Questions 1–16 are asked once for the OE and the same questions are asked for the EE (questions 17–32; Table 1).

**Table 1:** CBSEI-C32 items for the second stage of labor

*Number^a^*		English Items*	German Items**
*OE*	*EE*		
1	17	Relax my body.	Meinen Körper entspannen.
2	18	Get ready for each contraction.	Mich auf jede Wehe vorbereiten.
3	19	Use breathing during labor contractions.	Während der Wehe gezielt atmen.
4	20	Keep myself in control.	Mich beherrschen.
5	21	Think about relaxing.	An Entspannung denken.
6	22	Concentrate on an object in the room to distract myself.	Mich auf ein Objekt im Raum konzentrieren, um mich abzulenken.
7	23	Keep myself calm.	Mich ruhig halten.
8	24	Concentrate on thinking about the baby.	Meine Gendanken auf das Baby richten.
9	25	Stay on top of each contraction.	Jede Wehe meistern.
10	26	Think positive.	Positiv denken.
11	27	Not think about the pain.	Nicht an die Schmerzen denken.
12	28	Tell myself that I can do it.	Mir selber zureden, dass ich es schaffe.
13	29	Think about others in my family.	An andere Familienmitglieder denken.
14	30	Concentrate on getting though one contraction at a time.	Mich auf jede Wehe einzeln konzentrieren.
15	31	Focus on the person helping me in labor.	Meine Aufmerksamkeit auf die Person richten, die mir während der Geburt beisteht.
16	32	Listen to encouragement from the person helping me.	Auf die Ermutigung der Person hören, die mir hilft.

a Questionnaire number of the Outcome Expectancy (OE)/Efficacy Expectancy (EE).

* Ip et al.^5^ and ** Schmidt et al.^17^.

### German version of the CBSEI and CBSEI-C32

The German version of the CBSEI was translated by a midwifery graduate student in 2012. Psychometric properties of the CBSEI and CBSEI-C32 were tested with 123 nulliparas and 32 multiparas (n=155). The translation was carried out according to the high standards of the International Society for Pharmacoeconomics and Outcomes Research (ISPOR). Two forward and two backward translations were undertaken. The cross-cultural adaption was ensured by two native speakers^17,18^. As part of the development of the German version of the CBSEI, eight pregnant women participated in cognitive debriefing via a questionnaire in an antenatal class. They assigned scores ranging from 1 = ‘very good’ to 6 = ‘insufficient’ regarding the language and content intelligibility of items. The questionnaire also offered space for alternative suggestions for wording of the items. Some women took advantage of the opportunity and wrote down alternative formulations for two items. This means for example, for ‘An andere Familienmitglieder denken’ (original: ‘Think about others in my family’)^5^ a change into ‘An meine Familie denken’ (in English: ‘Think about my family’)was suggested (Schmidt G. et al., 2015^17^ and Zinsser LA, Stoll K, Gross MM, unpublished data, 2021). In the cognitive debriefing, three items (numbers: 3, 13 and 16 in the OE subscale) had a mean value over 2.0 points (calculated from the points 1 to 6), and one of them (item 16) was changed in its sentence structure: 'Auf die Ermutigung der Person, die mir hilft, hören' into 'Auf die Ermutigung der Person hören, die mir hilft'. For both questionnaires (CBSEI, CBSEI-C32), reliability and one-dimensionality could be confirmed. The short version of the CBSEI in particular, was perceived as user-friendly by participants.

In the current study^19^, it was considered whether the CBSEI-C32 should be included for the measurement of self-efficacy. During the pilot phase, possible difficulties in using the German version of the CBSEI-C32 became apparent. These are discussed in this article. Specifically, we explore whether some of the CBSEI items might need to be changed, in order to reflect contemporary conceptualizations of coping with labor and birth, and health-oriented statements, so that the items are easier to understand for childbearing women.

## METHODS

Convenience sampling was used to recruit nulliparas, who planned a natural birth, free from pharmacological or technological interventions, as part of a pilot project to improve childbirth preparation. Between 28+0 and 31+0 weeks of gestation, ten women completed the German short version of the CBSEI^19^. To evaluate how women felt about using the CBSEI-C32 tool and whether any questions were difficult to answer, two open-ended questions were added. Responses to these questions provided insight into the acceptability and comprehensibility of the German CBSEI-C32 among nulliparas in the pilot study and allowed us to assess whether participants understood the items and corresponding behavior being queried.

## RESULTS

### Sociodemographic characteristics

The pregnant women were aged 26–37 years and expecting their first child. All but one had 12 or 13 years of secondary education. All participants attended antenatal class.

### Acceptability of the German CBSEI-C32

In general, the inventory was positively rated. Six out of ten women (W1, W2, W3, W7, W9, W10) gave feedback that they found the CBSEI items good or interesting. Four women noted that completing the items helped them prepare for childbirth (W2, W3, W7, W9):

*‘Good, also as mental preparation for the birth.’* (W7)

The Likert scale with 1 to 10 points from the CBSEI was criticized by three women (W1, W3, W9). They wished to have a smaller range of response options, to make it easier to complete the scale.

### Comprehensibility of the German CBSEI-C32

Of the ten participants, six (W1, W3, W4, W5, W8, W9) reported problems with the comprehension of some questions.

With one item of the CBSEI-C32: Number 7 on the OE subscale, and on the EE subscale number 23, five participants (W3, W4, W5, W8, W9) had problems understanding the item ‘Keep myself calm’^5^ which was translated into ‘Mich ruhig halten’^17^. The word ‘calm’ in this context can be interpreted as meaning that the woman remains relaxed, rather than becomes nervous or upset, even in a difficult situation, which reflects coping on a cognitive and emotional level. The German translation could be back translated into: ‘Keep myself quiet’ (to make no or not much noise) and it could also be understood as: ‘Keep myself steady’ (not moving; firmly held in a particular position)^20^. While answering the items one woman asked:

*‘Keep calm in the sense of not moving?’* (W3)

While another participating woman asked:

*‘What does it mean: pull myself together or use techniques to calm me down and focus on breathing?’* (W9)

The meaning of the German translation of the item seemed to be difficult to understand and ambiguous as women interpreted the statement primarily as a physical not cognitive action.

A potential problem was also raised with item 4. The statement confused three women (W4, W8, W9). The original statement ‘Keep myself in control’^5^ can be interpreted as having the ability or power to behave as you want, for example, to remain calm in challenging situations. The German translation can be understood and interpreted in different ways with regard to women’s behavior during birth. ‘Mich beherrschen’^17^ can be also understood and back translated as ‘To contain yourself or to control strong feelings’^20^. Participants asked how they should interpret this statement. One woman asked:

*‘What does “beherrschen” (in English: control) mean: pull together, e.g. not screaming or vocal toning when I feel like it?’* (W9)

Another woman was wondering:

*‘Must I do that?’* (W4)

Six items (numbers: 1, 2, 5, 9, 11 and 15), were difficult to understand for one or two women. It was unclear what kind of action should be implemented based on the assessed statement, such as item 9 (‘Stay on top of each contraction’)^5^ or 15 (‘Focus on the person helping me in labor’)^5^. The participants made no suggestions for improvement of the items. Some items did not make sense to pregnant women in the pilot study, e.g. item number 1 in combination with number 2 (‘Relax my body’ and ‘Get ready for each contraction’). These items were seen as a contradiction, as well as number 5 (‘Think about relaxing’) and 11 (‘Not think about pain’)^5^. For these items, the language did not seem to be a problem, but the content itself and the coping behavior the items described were queried. One woman (W4) did not see the point to prepare for the next rhythmic contraction to come, but preferred instead to focus on the pauses between the contractions.

## DISCUSSION

Potential issues with the user-friendliness of the German version of the CBSEI were identified through pilot testing with ten first time mothers who completed the CBSEI-C32. The results also draw into question whether some items represent contemporary constructions of childbirth. The feedback from the participating women offers the opportunity to better understand how the items are understood by users and whether the statements of the CBSEI can be transferred to the behavior to be implemented for labor and birth. A rigorous translation process was chosen to minimize possible sources of error for the German version of the CBSEI. In the initial cognitive debriefing that was done in 2012, items 4 and 7 were not identified as problematic. They had low scores (<2.0) which means ‘good/B’ in school grades. There were neither too many missing values, nor were the average scores lower than for the other items. In the pilot study carried out in 2018, the statements of the women showed that items 4 and 7 from the German CBSEI-C32 could be interpreted and answered in different ways. It should be noted that there are no right or wrong answers to the CBSEI questionnaire, but rather the items asses one’s own estimation of abilities. But in order to measure childbirth self-efficacy reliably, a clear understanding of the items is important. According to Lowe^2^, the two items in question are central features of the construct of childbirth self-efficacy (relaxation, control). Women’s feedback on the general use of the questionnaire was positive in both studies (Zinsser LA et al., 2020^19^ and Zinsser LA, Stoll K, Gross MM, unpublished data, 2021), however, in the pilot study two participants did not complete the questionnaire due to comprehension problems (Zinsser LA, Stoll K, Gross MM, unpublished data, 2021).

The participating women and the authors are questioning if some items of the CBSEI measure contemporary constructions/views of childbirth self-efficacy. The tool itself is over 25 years old and much has changed during this time. For example, childbirth preparation is evolving, the role of partners is more prominent, and a higher value is placed on women making decisions about their own care^21^. Also, the kind of care that pregnant women experience has shifted more towards a health-oriented focus, as can be seen through the health literacy movement^22^ and the focus on keeping birth normal^23^. As mentioned in the results, one woman (W4) criticized the phrasing of the coping statements, and would have preferred a more health-oriented approach. Also, the feedback that was given about other items (1, 2, 5 and 11) indicates that some of the coping strategies for labor and birth did not resonate with the pregnant woman in the pilot study. It is possible that other aspects are more important nowadays to prepare for childbirth, and to acquire and increase childbirth self-efficacy. For example, midwifery science has identified that it is important to stay in the present moment and accept childbirth pain. An open, focused and accepting mind is helpful for successful coping; this appears to be a key concept^24,25^. Yet no statement in the CBSEI assesses this aspect. It should be considered whether some CBSEI items need to be revised or new items added, in consideration of current evidence on effective coping mechanisms. Because self-efficacy is a theoretically driven construct, the statements must address self-efficacy in labor and birth^1^. It might be worthwhile to use a modified expert review process, to assess the relevance and clarity of each CBSEI item with a contemporary diverse group of nulliparas and multiparas, and enable participants to identify missing items. This process might occur over several rounds, with women actively participating in revision of items deemed to be less relevant or clear. Generation of new items could also be informed via a systematic review of the literature about the concept of childbirth self-efficacy. These new items could then be assessed for clarity and relevance by either a diverse group of childbearing women or content experts, or both, and include assessment of related concepts such as confidence^26^. A concept analysis on supporting confidence of childbearing women was published in 2018^27^. Because the concept analysis is not solely focused on self-efficacy, it can only serve as an orientation to identify aspects that support self-efficacy in childbirth. For example, the finding from Neerland^27^, that women’s confidence is supported through a ‘safe environment’, is not reflected in the theory of self-efficacy^1^, in contrast to topics like knowledge or belief in the body’s innate ability to birth, that Neerland^27^ identified. We need to keep in mind that research for identifying individual coping strategies for dealing with labor and birth, especially in low intervention birth settings (home and birth center), can help refine the CBSEI. The CBSEI should be checked to identify which items could serve as the basis for a new questionnaire, because there are clear and appropriate coping strategies when dealing with labor pain, such as item 3 on breathing. These could be complemented with other coping behaviors that are positively worded and serve to empower rather than restrain women. However, aspects of empowerment are often not the focus of health programs^28^.

The statements from the women, about the 10-point Likert scale of the CBSEI items being too onerous, are understandable from the side of user-friendliness. From a scientific position, Bandura^29^ points out the importance of having a wider range of response options, to enable more nuanced assessment. Nevertheless, it is advisable to test the CBSEI with fewer response options, in order to simplify application of the scale. Internal consistency reliability of the scale is likely to still be high.

### Strengths and limitations

A strength of this study is that the participants were able to critically question the CBSEI statements and that feedback was similar. For example, 5 of 10 participants had difficulty understanding item 7. This consistency is reassuring and instills confidence in the findings. The German version of the short CBSEI was only tested with nulliparas because the planned intervention study was restricted to nulliparas. This is a strength of the study as nulliparas have no previous experience with childbirth and there is value in examining the reactions of nulliparas to the translated CBSEI items rather than conflating their responses with those of multiparas who might view the items in the context of previous experiences. The small sample size, although typical of pilot tests, must be seen as a limitation of the study as results based on larger sample sizes might have yielded additional feedback.

## CONCLUSIONS

The CBSEI-C32 is a valid tool and should be used as long as there is no alternative to measure childbirth self-efficacy. The German CBESEI-C32 should be tested without any changes to the items, because results from the pilot study described in this study are based on a small sample size. Future testing of the CBSEI should include nulliparas as well as multiparas, with different sociodemographic backgrounds. In order to be internationally comparable, adaptions are sometimes necessary but should be kept to a minimum.

## Data Availability

The data supporting this research is available from the authors on reasonable request.
